# Combinatorial genetic replenishments in myocardial and outflow tract tissues restore heart function in *tnnt2* mutant zebrafish

**DOI:** 10.1242/bio.046474

**Published:** 2019-12-09

**Authors:** Lian Liu, Fei Fei, Ranran Zhang, Fang Wu, Qian Yang, Feng Wang, Shaoyang Sun, Hui Zhao, Qiang Li, Lei Wang, Youhua Wang, Yonghao Gui, Xu Wang

**Affiliations:** 1 Department of Cardiology, Children's Hospital of Fudan University, Shanghai 201102, China; 2 Cancer Metabolism Laboratory, Cancer Institute, Fudan University Shanghai Cancer Center, Shanghai 200032, China; 3 Key Laboratory of Metabolism and Molecular Medicine, Ministry of Education, Department of Biochemistry and Molecular Biology, School of Basic Medical Sciences, Fudan University, Shanghai 230002, China; 4 Department of Pediatrics, the Affiliated Hospital of Qingdao University, Qingdao, Shangdong 266003, China; 5 Longhua Hospital, Shanghai University of Traditional Chinese Medicine, Shanghai 200032, China; 6 Translational Medical Center for Development and Disease, Shanghai Key Laboratory of Birth Defect, Institute of Pediatrics, Children's Hospital of Fudan University, Shanghai 201102, China

**Keywords:** Zebrafish, Cardiac troponin T (TNNT2), Conditional transgene, Genetic replenishment, Dilated cardiomyopathy (DCM), Outflow tract (OFT), Cardiomyocyte

## Abstract

Cardiac muscle troponin T (Tnnt2) mediates muscle contraction in response to calcium ion dynamics, and *Tnnt2* mutations are associated with multiple types of cardiomyopathy. Here, we employed a zebrafish model to investigate the genetic replenishment strategies of using conditional and inducible promoters to rescue the deficiencies in the heart. *tnnt2a* mutations were induced in zebrafish via the CRISPR/Cas9 technique, and the mutants displayed heart arrest and dilated cardiomyopathy-like phenotypes. We first utilized the classic myocardial promoter of the *myl7* and *TetOn* inducible system to restore *tnnt2a* expression in myocardial tissue in *tnnt2a* mutant zebrafish. However, this attempt failed to recover normal heart function and circulation, although heart pumping was partially restored. Further analyses via both RNA-seq and immunofluorescence indicated that Tnnt2a, which was also expressed in a novel group of *myl7*-negative smooth muscle cells on the outflow tract (OFT), was indispensably responsible for the normal mechanical dynamics of OFT. Lastly, *tnnt2* expression induced by OFT cells in addition to the myocardial cells successfully rescued heart function and circulation in *tnnt2a* mutant zebrafish. Together, our results reveal the significance of OFT expression of Tnnt2 for cardiac function and demonstrate zebrafish larva as a powerful and convenient *in vivo* platform for studying cardiomyopathy and the relevant therapeutic strategies.

## Abstract (precis)

**Summary:** Tnnt2 expression in the outflow tract is essential for cardiac function, and zebrafish is a powerful *in vivo* model for investigating cardiomyopathy genetics.

## INTRODUCTION

Dilated cardiomyopathy (DCM) and hypertrophic cardiomyopathy (HCM) are two common cardiomyopathies with an estimated prevalence of 1:250 and 1:200 in adults (McKenna et al., 2017; Prondzynski et al., 2018). The prognoses of cardiomyopathies are poor, and the annual mortalities for DCM and HCM are 1.55% and 2.5%, respectively (Pelliccia et al., 2017; Vischer et al., 2009). During the past few decades, genetic screening has identified a list of autosomal inherited mutations as potential ‘drivers’ or susceptible factors for cardiomyopathy, and approximately 40–50% of all cases can be attributed to genetic mutations (Zou et al., 2013; Paldino et al., 2018). Among these, genes coding sarcomere proteins are frequently found to be dysfunctional in both DCM and HCM, and it is estimated that around 30–40% DCM-associated mutations and 60% HCM-associated mutations occur in sarcomere genes (McNally et al., 2013; Hershberger et al., 2013; Veselka et al., 2017). Besides, mutations in a single sarcomere gene like cardiac troponin T (*TNNT2*) are sufficient to cause cardiomyopathy, and *TNNT2* mutations are the most common ‘drivers’ of thin filament deficiency in both DCM and HCM (Hershberger et al., 2013; Veselka et al., 2017). Most human *TNNT2* mutations are located in central and C-terminal domains of cardiac troponin T and are responsible for both familial cardiomyopathy and sporadic cardiomyopathy (Forissier et al., 1996; Van Driest, 2003; McConnell et al., 2017).

Due to their transparency and small size, zebrafish have been extensively used to investigate the genetics of heart development and to model the pathogenesis of cardiomyopathy *in vivo*. In zebrafish, a total of four *tnnt2a* mutations have been previously reported: *tnnt2a^tc300b^*, *tnnt2a^b109^*, *tnnt2a^ex5Δ23^* and *tnnt2a^ex10Δ2^*. The former two, *tnnt2a^tc300b^* and *tnnt2a^b109^*, were generated by ENU or γ-ray mutagenesis followed by phenotype screening (Sehnert et al., 2002). The *tnnt2a^tc300b^* mutant carries a point mutation in the splice site at the intron2-exon3 junction, causing a frameshift and a premature stop in the central domain, while *tnnt2a^b109^* bears a 13-bp deletion of the 5′ regulatory region and displays similar patterns (Sehnert et al., 2002). Contrastingly, the latter two, *tnnt2a^ex5Δ23^* and *tnnt2a^ex10Δ2^* were both generated by our group, carrying a 2-bp deletion in the C-terminal domain and a 23-bp deletion in the central domain, separately (Liu et al., 2017). Although both HCM (Gilda et al., 2016) and DCM (Rani et al., 2014) have been found in patients with *TNNT2* mutations, all four zebrafish mutants seem to display only DCM-like phenotypes with atrium and ventricle enlargement (Sehnert et al., 2002; Liu et al., 2017). Moreover, electron microscopy in our previous studies as well as in other studies has shown that thin filament formation does not occur in those mutants (Sehnert et al., 2002; Liu et al., 2017). In summary, *tnnt2* mutant zebrafish display early onset and highly consistent phenotypes and serve as a specialized but convenient DCM model for assessing potential therapeutic strategies.

In this study, we investigated the transgenic replenishment strategies targeting myocardial and non-myocardial tissues in the zebrafish DCM model to find whether manipulations in these tissues are sufficient for full recovery of heart function. We first demonstrated that *tnnt2a* mutant zebrafish have typical DCM-like phenotypes. We then designed a *myl7* promoter-driven and Tet-On inducible transgenic cascade to ectopically express *tnnt2a* mRNA only in cardiomyocytes of the mutants. However, we failed to restore mechanical behaviour of the outflow tract (OFT). The results of gene expression and microscopy indicate that *tnnt2a* was also expressed in a small proportion of the *myl7*-negative smooth muscle on the OFT, and the additional *tnnt2a* supplements in these cells significantly rescued the dynamics of the OFT and recovered heart function. To conclude, we have identified a novel non-myocardial Tnnt2+ cell population that is indispensable to a functional OFT and will provide novel insights into therapeutic strategies against TNNT2 for rescuing heart deficiencies.

## RESULTS

### Generation and phenotype analyses of *tnnt2* mutant zebrafish

**Fig. 1.**
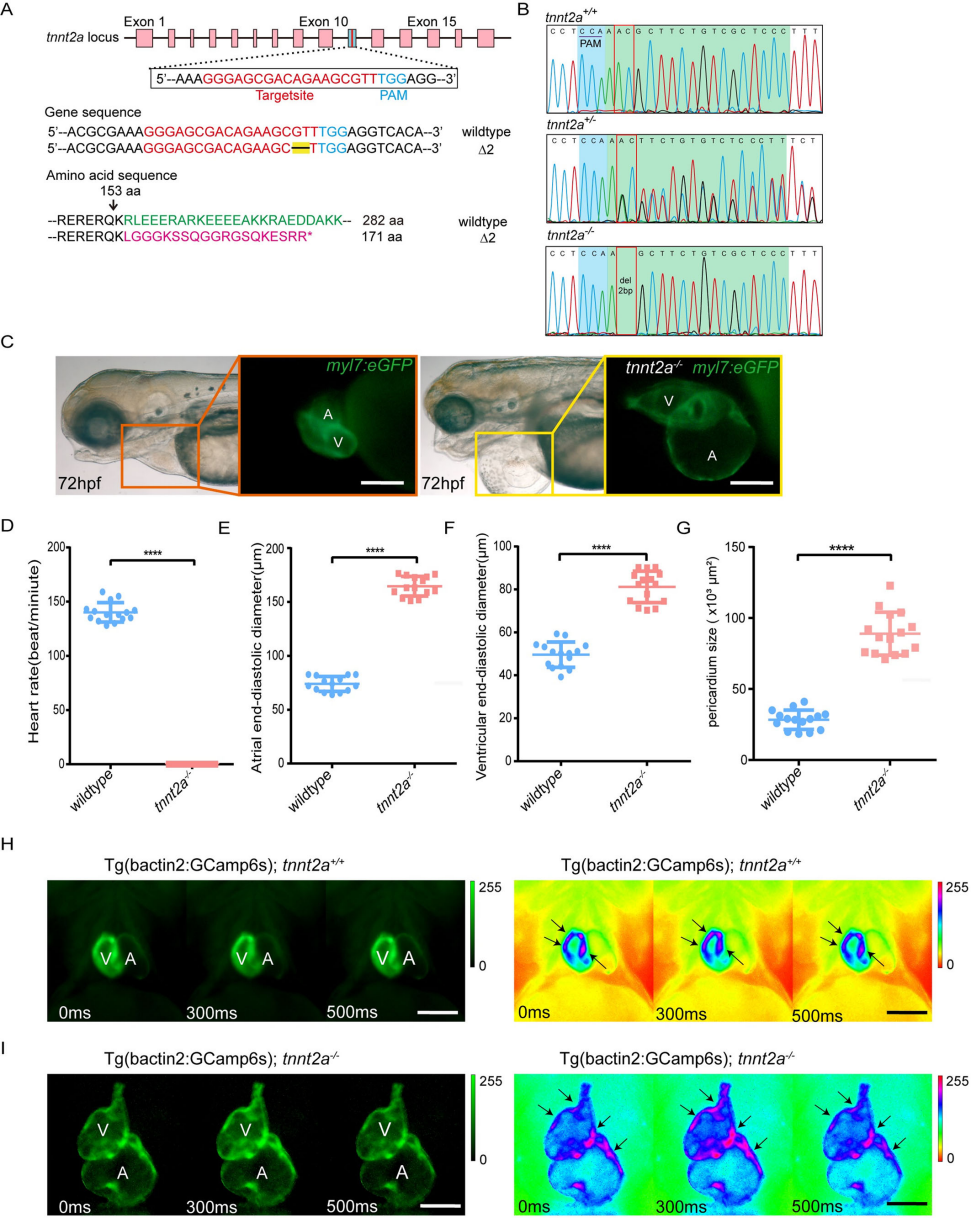
**Generation of *tnnt2a* mutant zebrafish and** **phenotype analyses.** (A) Graphical representation of the target site of *tnnt2a* for CRISPR/Cas9 gRNA and altered amino acid sequence of Tnnt2a of mutant zebrafish. (B) Genotype sequencing results of *tnnt2a*^+/+^ and *tnnt2a*^−/−^ zebrafish. (C) Representative images of atrial (A) and ventricular (V) enlargement, and pericardial effusion in 72 hpf *tnnt2a*^−/−^ zebrafish. Scale bars: 150 µm. (D–G) Statistics for the heart rate, atrial and ventricular diameter, and pericardium size in wild-type and *tnnt2a*^−/−^ zebrafish; ****P<0.0001; *n*=15 per group. Statistical differences were evaluated using two-tailed unpaired *t*-tests (D–F) or unpaired *t*-test with Welch's correction (G). (H,I) Fluorescent images and pseudo-colours of electrocardiac rhythm in Tg(*bactin2:GCamp6s*; *tnnt2a*^+/+^) and Tg(*bactin2:GCamp6s*; *tnnt2a*^−/−^) zebrafish; arrows represent increased Ca^2+^ flux in cardiomyocytes. The direction of Ca^2+^ flux was from venous to arterial end both in *tnnt2a*^+/+^ and *tnnt2a*^−/−^. Scale bars: 100 µm.

*Tnnt2a* mutant zebrafish were successfully generated via the CRISPR/Cas9 technique (Liu et al., 2017; Wang et al., 2018). Genotyping indicated that two bases of exon ten of *tnnt2a* were deleted as previously reported (Fig. 1A,B). The frame shift mutation induced a protein-truncating variant, and the homologous *tnnt2a^ex10Δ2^* mutant (*tnnt2a^−/−^*) displayed dilated cardiomyopathy-like phenotypes, such as heart arrest as well as atrium and ventricle enlargement 24 h post fertilization (hpf) (Fig. 1C–F). Meanwhile, mutant zebrafish also demonstrated significant pericardial effusion, a phenotype of heart failure, at approximately 72 hpf (Fig. 1C,G). To determine whether the cardiomyocytes in mutant zebrafish maintained normal electrocardiac rhythm, we introduced the Tg(*bactin2:GCamp6s*) transgenic background that expresses a slower version of fluorescent calcium sensor ubiquitously (Chen et al., 2013). In the completely arrested mutant hearts at 72 hpf, a fluorescent wave of Ca^2+^ influx into cardiomyocytes from the venous to arterial end could still be observed (Fig. 1H,I; Movies 1 and 2). This confirmed that *tnnt2a* mutation blocks the electrical-to-mechanical signaling transduction in cardiomyocytes. In addition, the heterozygous *tnnt2a^ex10Δ2^* (*tnnt2a^+/−^*) did not display any defects in heart function at all stages as previously reported (Liu et al., 2017).

### Heart function is not rescued in Tg(*myl7:tetOn; tre:tnnt2a-p2a-mKate2; myl7:eGFP*); *tnnt2a*^−/−^ zebrafish

**Fig. 2.**
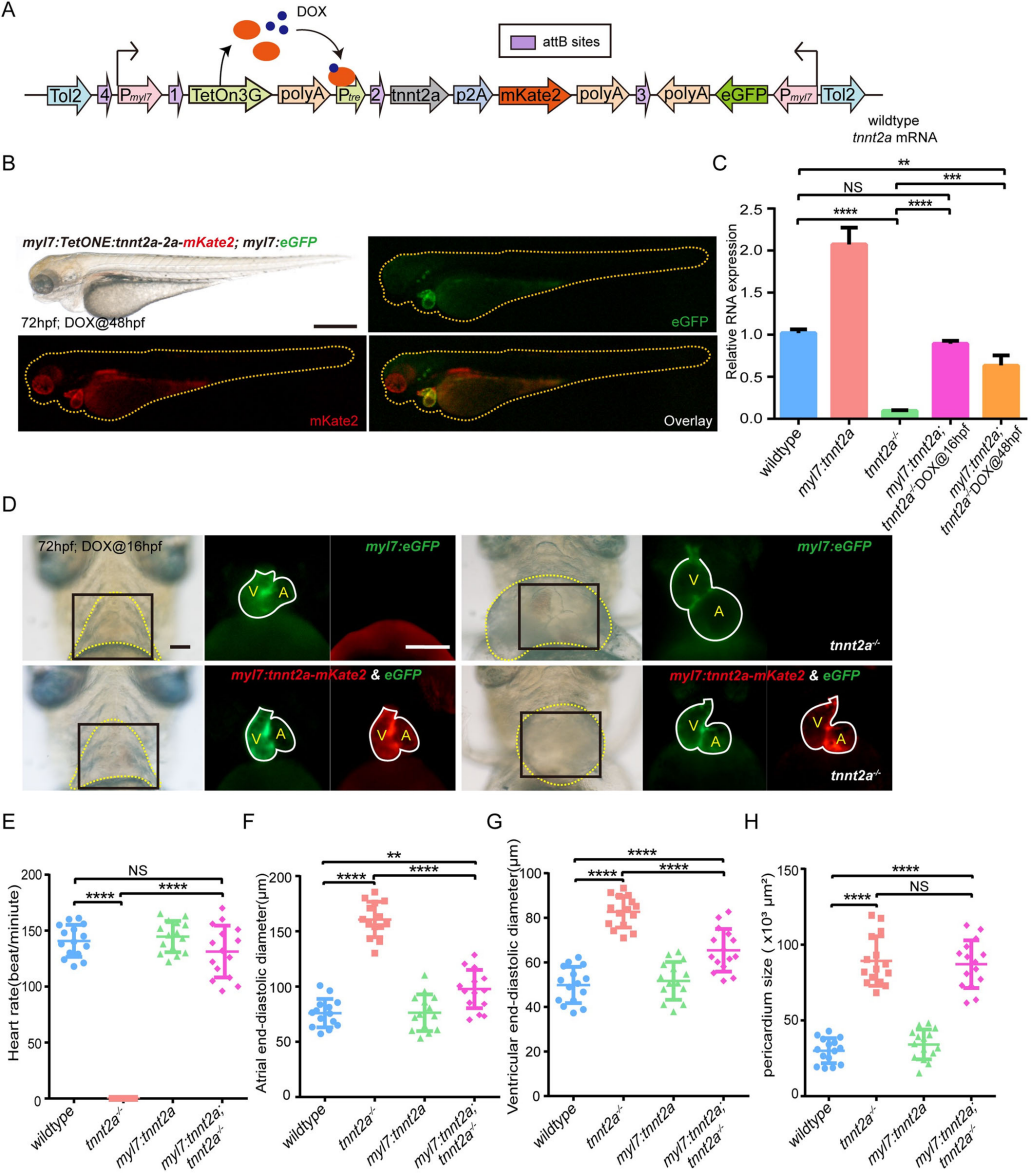
**Myocardial-specific and DOX-inducible *tnnt2a* expression fail to rescue heart function in *tnnt2a* mutant zebrafish.** (A) Structure of the tol2-flanked transgenic vector. DOX, doxytetracycline. (B) Lateral views of a 72 hpf transgenic zebrafish after DOX induction at 48 hpf in green and red fluorescence. Scale bar: 300 µm. (C) Real-time RT-PCR of wild-type form of *tnnt2a* cDNA in wild-type, *tnnt2a^−/−^*, Tg(*myl7:tetOn*; *tre:tnnt2a-p2a-mKate2*; *myl7:eGFP*) [Tg(*myl7:tnnt2a*)] following addition of DOX at 16 hpf, and Tg(*myl7:tetOn*; *tre:tnnt2a-p2A-mKate2*; *myl7:eGFP*; *tnnt2a^−/−^*) [Tg(*myl7:tnnt2a*); *tnnt2a^−/−^*] following addition of DOX at 16 or 48 hpf. **P<0.01, ***P<0.001, ****P<0.0001, NS, not significant; *n*=30 per group. Statistical differences were evaluated using two-way ANOVA. (D) Representative images of hearts of four groups from ventral view. Yellow dotted line, pericardium; white line, heart outline; black box, area of the fluorescent images; V, ventricle; A, atrium. Scale bar: (bright field) 50 µm, (fluorescent) 100 µm. DOX was added at 16 hpf. (E–H) Statistics for heart rate, atrial and ventricular end-diastolic diameter, and pericardium size in four groups; **P<0.01, ****P<0.0001, NS, not significant; *n*=15 per group. Statistical differences were evaluated using Kruskal–Wallis test (E) or one-way ANOVA (F–H).

To perform myocardial-specific gene replenishment, we selected the promoter of *myosin light 7* (*myl7*), which is shorter than the reported *tnnt2a* promoter (Verma and Parnaik, 2017). *Myl7* promoter has also been extensively employed to drive gene expression in atrial and ventricular myocardium in zebrafish hearts at all stages (Thisse et al., 2004; Hsiao et al., 2003). We generated *myl7* promotor-driven and dosage-induced (Tet-On system) transgenic cassettes (Yao et al., 2018) for delicate regulations of *tnnt2a* expression (Fig. 2A,B). Afterwards, *tnnt2a^−/−^* mutant background was introduced into the transgenic lineage by out-crossing twice to generate Tg(*myl7:tetOn; tre:tnnt2a-p2a-mKate2; myl7:eGFP*); *tnnt2a*^−/−^ embryos. Doxytetracycline (DOX) was added before or after the initial time of cardiac contraction (21–22 hpf) to determine whether the genetic rescue was time sensitive. Real-time RT-PCR on dissected hearts demonstrated that DOX administrated before cardiac contraction produced a higher level of *tnnt2a* mRNA, equivalent to the level in the wild-type hearts (Fig. 2C). Fluorescent images also showed that homozygous mutant with enlarged atria and ventricle relieved abnormal heart phenotypes after adding DOX before the staining point of heartbeat (Fig. 2D). However, phenotyping and statistical analyses indicated that, although the heart beating was restored, and the dilated atrium/ventricle recovered partially (Fig. 2E–G), pericardium effusion remained, and pumping of the heart had barely improved blood circulation (Fig. 2H; Movie 3).

### OFT dysfunction is responsible for heart failure in Tg(*myl7:tetOn; tre:tnnt2a-p2A-mKate2*); *tnnt2a*^−/−^ zebrafish

**Fig. 3.**
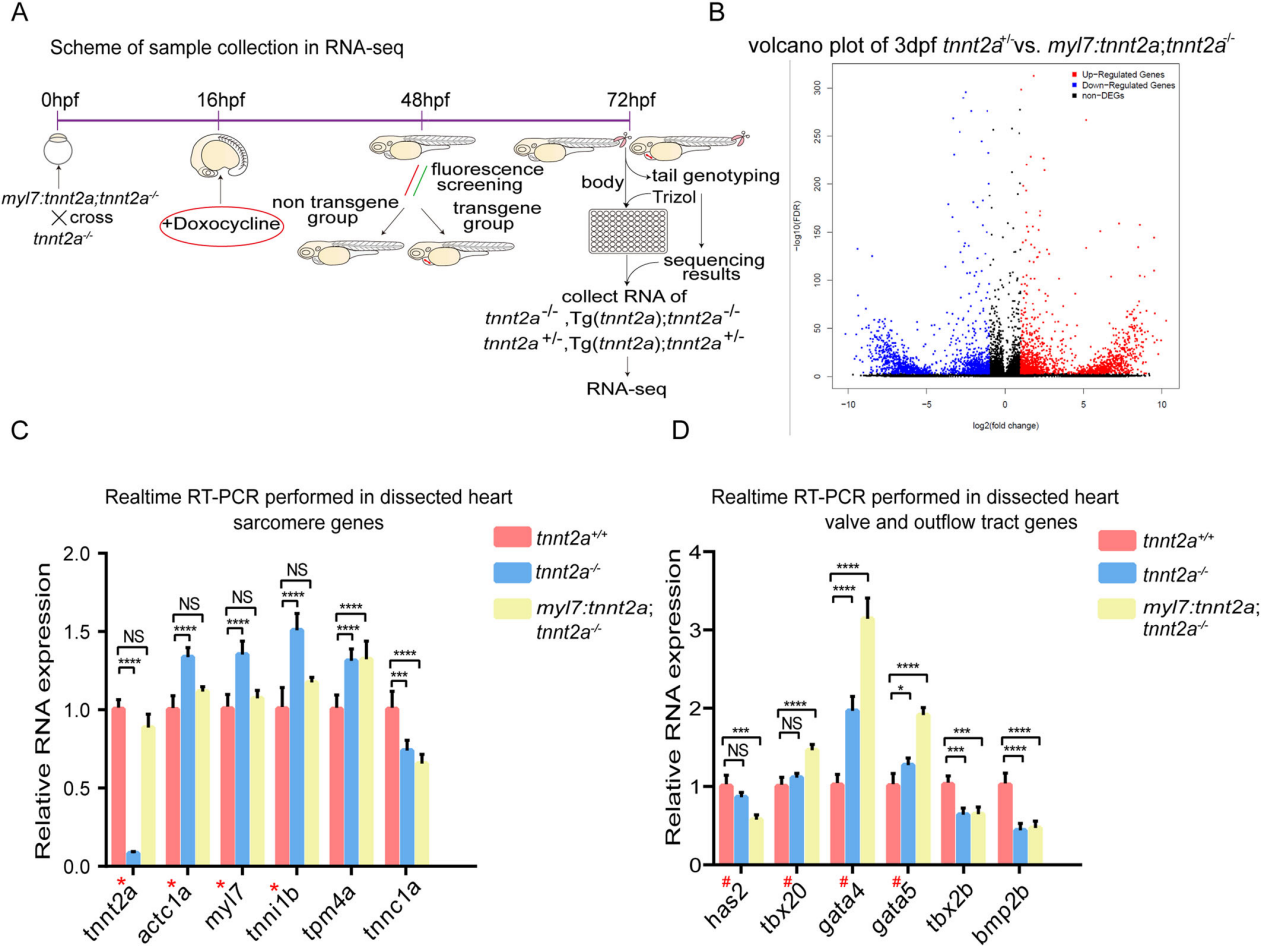
**Gene expression in OFT is deregulated in *tnnt2a* mutant zebrafish with myocardial *tnnt2a* supplementation.** (A) Workflow of sample collections for whole-fish RNA-seq of four groups: *tnnt2^+/−^*, *tnnt2a^−/−^* Tg(*myl7:tnnt2a*); *tnnt2a^−/−^* and Tg(*myl7:tnnt2a*); *tnnt2a^+/−^*. (B) Volcano plot of differentially expressed genes in *tnnt2a*^+/−^ versus Tg(*myl7:tnnt2a*); *tnnt2a^−/−^*. (C,D) Sarcomere- and OFT-relevant gene expression in dissected heart of *tnnt2a*^+/+^, *tnnt2a*^−/−^, and Tg(*myl7:tnnt2a*); *tnnt2a^−/−^* after DOX induction at 16 hpf. *P<0.05, ***P<0.001, ****P<0.0001, NS, not significant; *n*=30 per group. Statistical differences were evaluated using two-way ANOVA. Red asterisks, sarcomere genes in Tg(*myl7:tnnt2a*); *tnnt2a^−/−^* can almost return to wild-type level after DOX induction; red hashes, OFT-relevant genes in Tg(*myl7:tnnt2a*); *tnnt2a*^−/−^ are more deviated from wild-type level after DOX induction compared with *tnnt2a^−/−^*.

To identify the reason that myocardial-specific gene replenishment failed to rescue the heart function in the mutant zebrafish, we performed a transcriptome analysis to obtain some hints about further investigation. Since the larval hearts are very small in size and it is difficult to gather sufficient RNA samples from dissected hearts, we first performed a whole-fish RNA-seq in four sibling groups: *tnnt2a*^+/−^, *tnnt2a*^−/−^, Tg(*myl7:tetOn; tre:tnnt2a-p2a-mKate2; myl7:eGFP*); *tnnt2a*^+/−^, and Tg(*myl7:tetOn; tre:tnnt2a-p2a-mKate2; myl7:eGFP*); *tnnt2a*^−/−^ at 3 dpf, and validated the findings via real-time RT-PCR on dissected heart tissues. The general distribution of differentially expressed genes (DEGs) in RNA-seq assays among all the four groups was displayed (Fig. 3A,B; Fig. S1A,B and Tables S1–S3). In general, the whole-mount expressions of several sarcomere genes, *tnnt2a*, *actc1a*, *myl7* and *tnni1b,* in Tg(*myl7:tetOn; tre:tnnt2a-p2a-mKate2; myl7:eGFP*); *tnnt2a*^−/−^ zebrafish were recovered to a level similar to that of *tnnt2a*^+/−^, which was confirmed by real-time RT-PCR in dissected hearts at 3 dpf (Fig. 3C). However, the expressions of several genes relevant to valves and in OFT (Just et al., 2016; Laforest and Nemer, 2011; Cai et al., 2013; Li et al., 2016), including *has2*, *tbx20*, *gata4*, *gata5*, *tbx2b* and *bmp2b*, were deregulated in the dissected hearts of Tg(*myl7:tetOn; tre:tnnt2a-p2a-mKate2; myl7:eGFP*); *tnnt2a*^−/−^, and expressions of *has2*, *tbx20*, *gata4* and *gata5* in the dissected hearts of Tg(*myl7:tetOn; tre:tnnt2a-p2a-mKate2; myl7:eGFP*); *tnnt2a*^−/−^ were further deregulated to an even worse degree than those of *tnnt2a*^−/−^ (Fig. 3D).

**Fig. 4.**
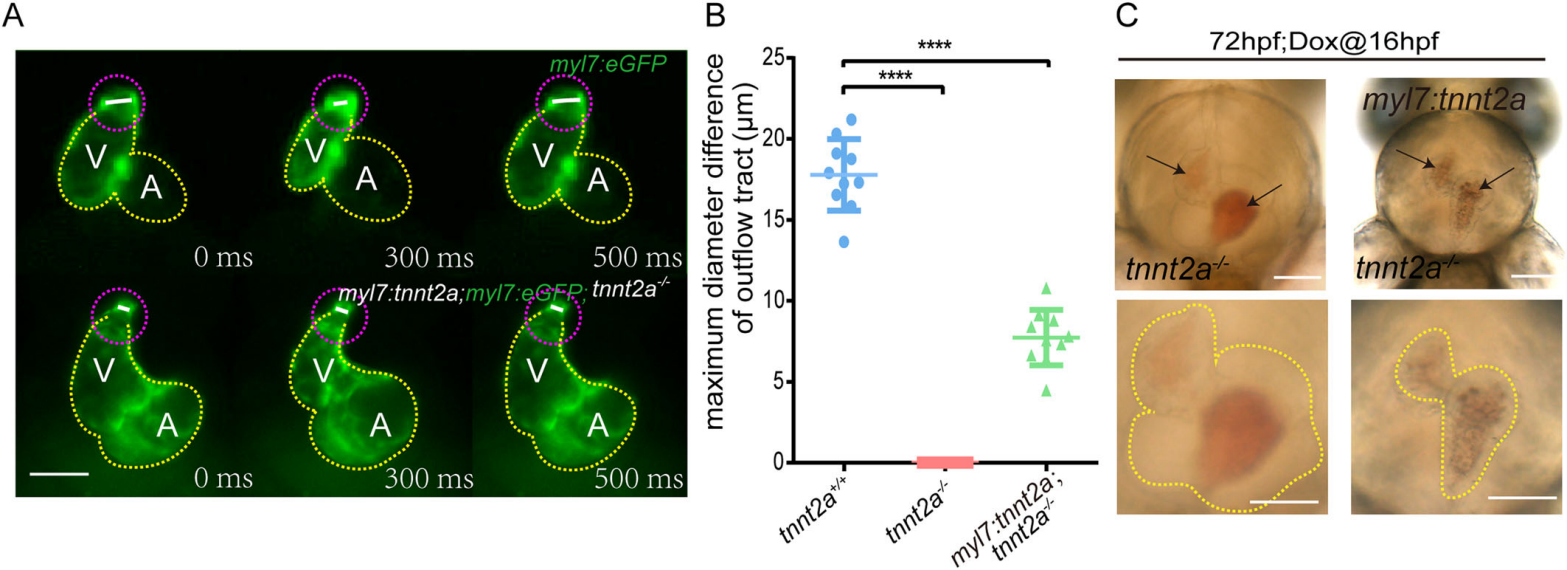
**Mechanical behaviours of OFT are deregulated in *tnnt2a* mutant zebrafish with myocardial *tnnt2a* supplementation.** (A) Representative images of the OFT morphologies at systole and diastole stages of a cardiac cycle. V, ventricle; A, atrium; yellow dotted line, heart outline; white line, OFT diameter; purple dotted line, OFT outline. Scale bar: 100 µm. (B) Statistics of maximum diameter difference of OFT in *tnnt2a*^+/+^, *tnnt2a*^−/−^ and Tg(*myl7:tnnt2a*); *tnnt2a^−/−^*. ****P<0.0001; *n*=30 per group. Statistical differences were evaluated using Kruskal–Wallis test. (C) Images of the stacked blood cells in the heart chambers of *tnnt2a*^−/−^ and Tg(*myl7:tnnt2a*); *tnnt2a^−/−^* zebrafish at 72 hpf. Arrows show the stacked red blood cells. Scale bars: 100 µm.

Consistent with the gene expression evidence, the mechanical contraction of the OFT in Tg(*myl7:tetOn; tre:tnnt2a-p2a-mKate2; myl7:eGFP*); *tnnt2a*^−/−^ was dysfunctional during the cardiac cycle (Movie 4), and it was observed that the maximum diameter of OFT in Tg(*myl7:tetOn; tre:tnnt2a-p2a-mKate2; myl7:eGFP*); *tnnt2a*^−/−^ was significantly reduced with restricted movement range compared with that of the normal OFT (Fig. 4A,B). Furthermore, similar to *tnnt2a*^−/−^, blood cells in the heart chambers of Tg(*myl7:tetOn; tre:tnnt2a-p2a-mKate2; myl7:eGFP*); *tnnt2a*^−/−^ were mostly stacked and heart beating was restored (Fig. 4C). These results suggest that OFT dysfunction may be responsible for the unsuccessful rescue of heart function and blood circulation in Tg(*myl7:tetOn; tre:tnnt2a-p2-mKate2*); *tnnt2a*^−/−^.

### Additional replenishment of *tnnt2a* in the smooth muscle cells of OFT recovered the heart function of Tg(*myl7:tetOn; tre:tnnt2a-p2a-mKate2*); *tnnt2a*^−/−^ zebrafish

**Fig. 5.**
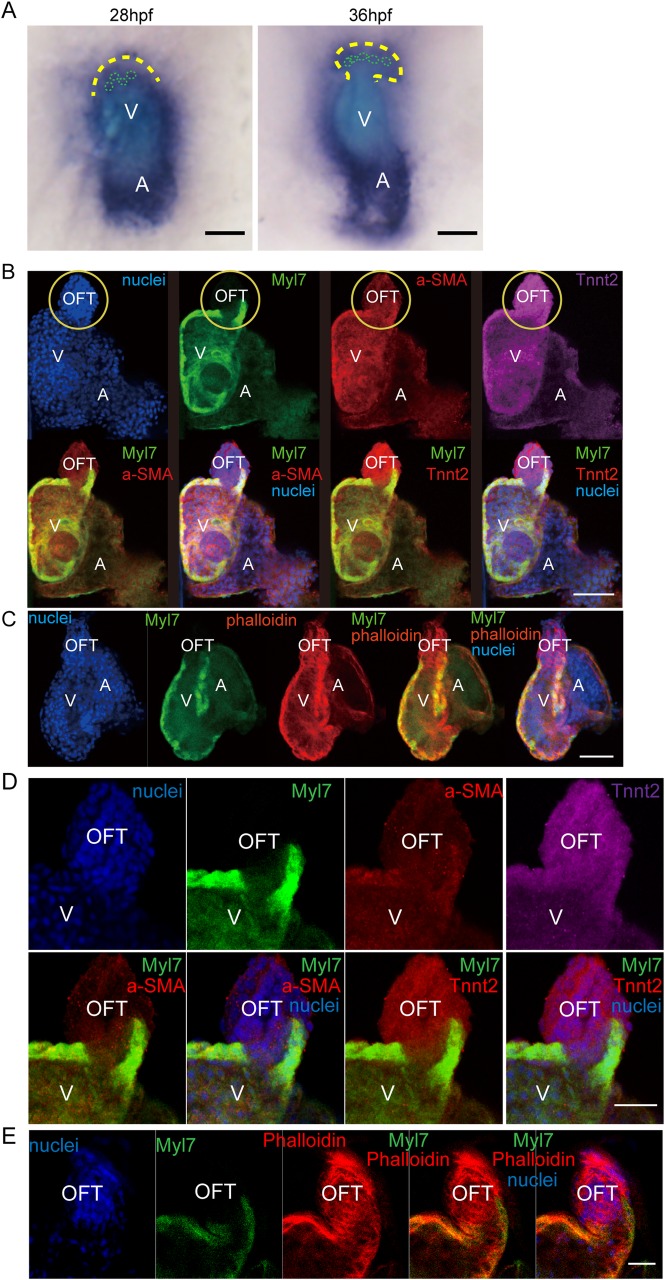
**Identification of the TNNT2-positive, Myl7-negative smooth muscle cells in the OFT.** (A) Images of *in situ* hybridization with tnnt2a anti-sense probe. Yellow dotted line, outflow tract outline; green dotted line, cell outline. (B) Immunofluorescence of Tnnt2, Myl7, and α-SMA staining in wild-type zebrafish hearts at 5 dpf. Nuclei is stained by Hoechst 33342. Yellow circle shows OFT area. (C) Myl7 zebrafish heart stained by phalloidin (F-actin) at 5 dpf. Nuclei is stained by Hoechst 33342. Scale bar: 150 µm. (D,E) Images of a single plane of Tnnt2, Myl7, α-SMA and phalloidin immunofluorescence staining. V, ventricle; A, atrium. Scale bars: (A–C) 150 µm, (D,E) 50 µm.

To further confirm whether *Tnnt2* was expressed in certain non-cardiomyocyte tissues of OFT, we first conducted *in situ* hybridization with a *tnnt2a* anti-sense probe and it was verified that *Tnnt2* was expressed in OFT (Fig. 5A). Then we performed immunofluorescence studies and revealed that a cluster of *myl7*-negative, Tnnt2-positive cells in cardiac OFT were stained by F-actin and α-smooth muscle actin (α-SMA) (Fig. 5B–E; Fig. S2), indicating that Tnnt2 is expressed in not only myocardial tissue, but also in some smooth muscles in the OFT. Smooth muscle cells are also present in the aortic valves of mammalian OFTs (Bairati and DeBiasi, 1981; Rogers et al., 2003), suggesting that these non-cardiomyocyte Tnnt2-positive smooth muscle cells may be evolutionally conserved between fish and mammals.

**Fig. 6.**
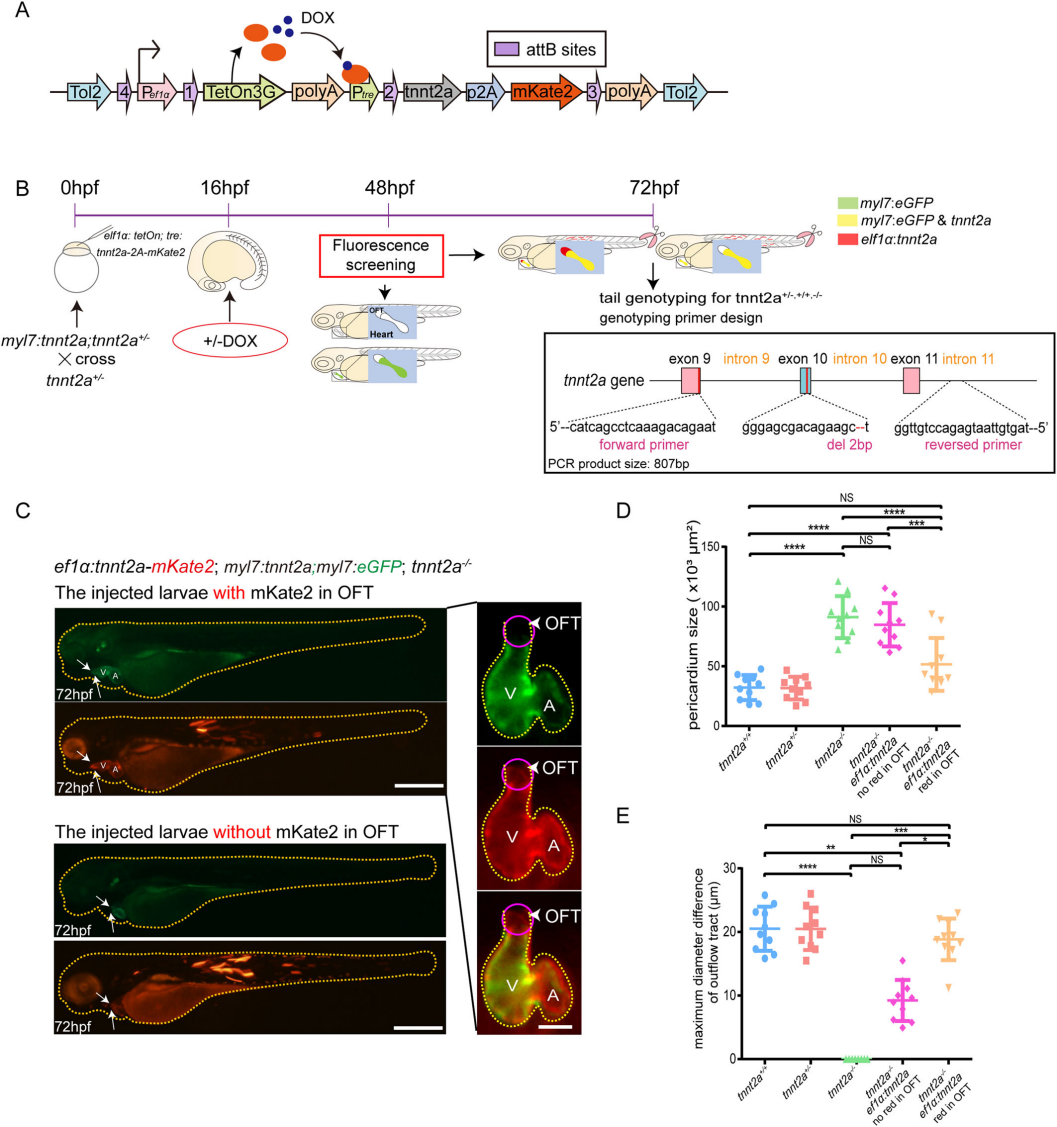
**Replenishments of *tnnt2a* in both myocardial cells and OFT significantly recover the heart function of tnnt2a mutant zebrafish.** (A) Structure of the transgenic vector for rescue injection. (B) Schematic workflow of the double-rescue experiments and phenotyping/genotyping. (C) Left: representative images of Tg(*myl7:tnnt2a*); *tnnt2a^−/−^* zebrafish injected by *eflα:tetOn; tre:tnnt2a-p2A-mKate2* [*eflα:tnnt2a*] with and without red fluorescence in OFT. Right: representative images of Tg(*myl7:tnnt2a*); *tnnt2a^−/−^* with additional *eflα:tnnt2a* in OFT. Yellow dotted lines, whole larvae or heart outlines; arrows and purple circle, mKate2-positive and eGFP-negative regions; V, ventricle; A, atrium; arrows and arrowheads, outflow tract. Scale bars: (lateral view) 200 µm, (ventral view) 150 µm. (D,E) Statistics of the maximum diameter difference of the OFT and pericardium size in *tnnt2a*^+/+^, *tnnt2a*^+/−^, *tnnt2a*^−/−^, Tg(*myl7:tnnt2a*); *tnnt2a^−/−^* injected by *eflα:tetOn; tre:tnnt2a-p2A-mKate2* [*eflα:tnnt2a*] with and without red fluorescence in OFT. *P<0.05, **P<0.01, ***P<0.001, ****P<0.0001, NS, not significant; *n*=30 per group. Statistical differences were evaluated using one-way ANOVA (D) and Kruskal–Wallis test (E).

Next, to validate that *Tnnt2* expression in the smooth muscle cell group was indispensable for normal functional OFT, we performed a double-rescue experiment on *tnnt2a*^−/−^ zebrafish by injecting *tol2* mRNA and the *elf1α:tetOn; tre:tnnt2a-p2a-mKate2* vector flanked by *tol2* repeats into Tg(*myl7:tetOn; tre:tnnt2a-p2a-mKate2; myl7:eGFP*); *tnnt2a*^−/−^embryos at the single-cell stage (Fig. 6A,B). The universal promoter *elf1α* was employed to induce mosaic expression in tissues including the OFT smooth muscle cell group because there is no available promoter for those OFT smooth muscle cells. The injected Tg(*myl7:tetOn; tre:tnnt2a-p2a-mKate2; myl7:eGFP*); *tnnt2a*^−/−^ larvae carrying additional red fluorescence of mKate2 (and Tnnt2a) around the OFT (without overlapping green fluorescence) were regarded as the double-rescued samples (Fig. 6C). Representative samples with and without additional mKate2 in the OFT of those injected larvae are shown in Fig. 6C, which indicates the successful/unsuccessful replenishment of *tnnt2a* in the specific smooth muscle cell group of OFT. In a typical larva with replenishment of *tnnt2a* in both cardiomyocyte and OFT, the heart contraction and OFT dynamics were both restored (labelled in red fluorescence), and blood circulation was smoothly recovered (Movies 5 and 6). The maximum diameter of the OFT and the size of the pericardium were also measured and statistical analysis showed that the heart of the injected Tg(*myl7:tetOn; tre:tnnt2a-p2a-mKate2; myl7:eGFP*); *tnnt2a*^−/−^ zebrafish with *tnnt2a-2A-mKate2* expressed in OFT performed better than those without *tnnt2a-2A-mKate2* expression in terms of pericardium size and OFT mechanic behaviour (Fig. 6D,E).

**Fig. 7.**
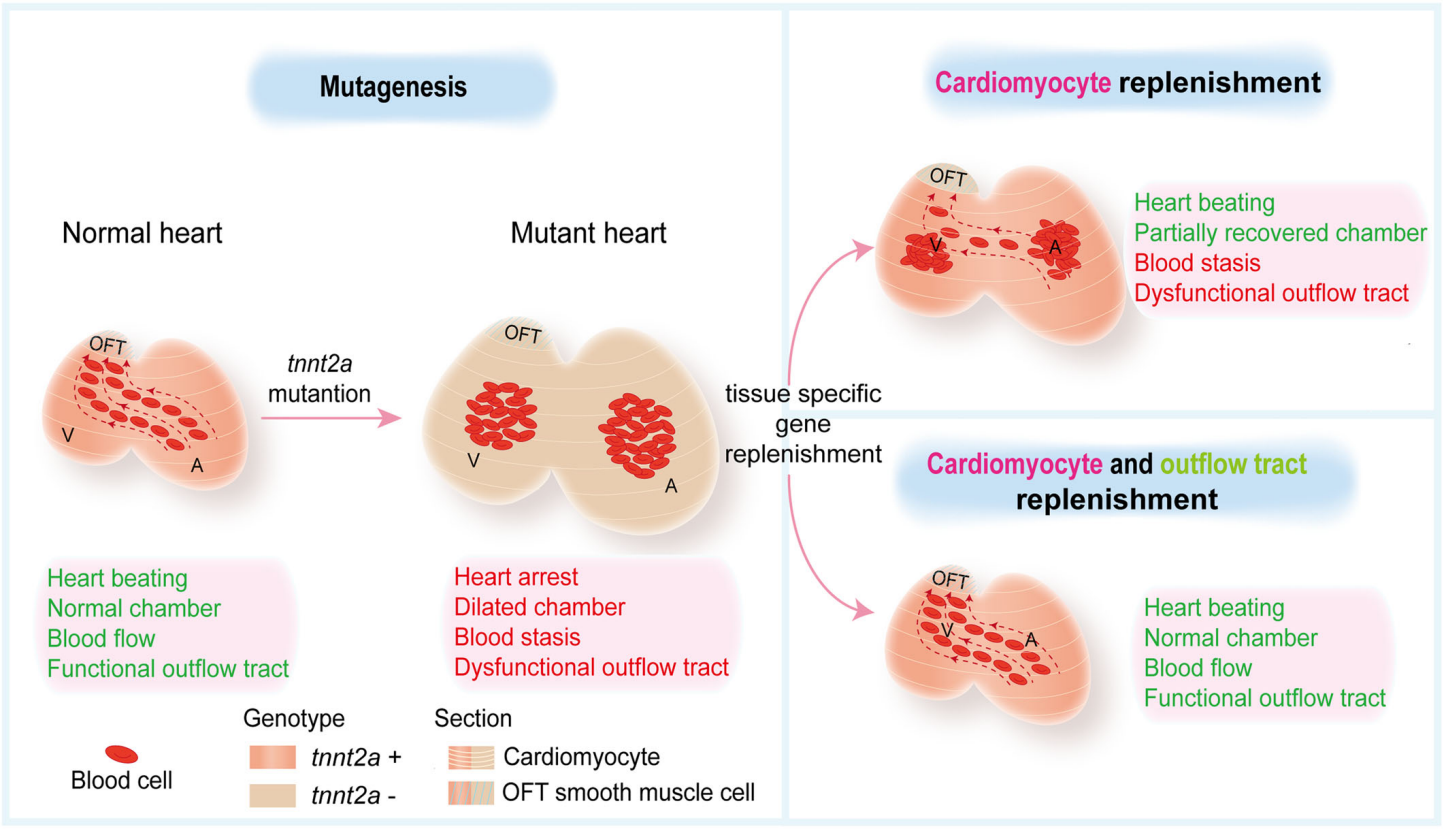
**Graphical abstract.** Myocardial-specific genetic replenishments are insufficient to rescue *tnnt2* mutant heart function, and combinatorial genetic replenishments in myocardial and OFT tissues restore heart function in *tnnt2* mutant zebrafish.

Together, our results reveal the importance of OFT expression of Tnnt2 for cardiac function and indicate the complexity of assessing therapeutic strategies for cardiomyopathy caused by sarcomere-based mutations (Fig. 7). Our study also demonstrates zebrafish larva as a powerful and convenient *in vivo* platform for modelling and investigating cardiomyopathy.

## DISCUSSION

### Use of zebrafish *tnnt2* mutants as highly specialized DCM models

Unlike the severe DCM-like phenotypes in the homozygous *tnnt2* mutant zebrafish, DCM caused by heterozygous (dominant) mutations in Tnnt2 is a progressive disease in human patients and usually occurs in adults (Pelliccia et al., 2017; Vischer et al., 2009). Hypothetically, the *tnnt2* heterozygous mutant and other non-shift mutants may better mimic adult patients. However, we did not observe any heart failure-like phenotypes in heterozygous mutants and other non-shift mutants up to 12 mpf (months post fertilization) (data not shown). The *tnnt2* homozygous mutant used in this study has a very early-onset phenotype and can only be considered as a highly specialized embryonic/larval model of DCM. In fact, the original purpose of our project was to integrate the inducible transgene into the mutant background to delay/adjust the onset of the DCM-like phenotypes at different stages (embryonic, larval, juvenile and adult stages). For that purpose, the *tnnt2* promoter instead of the *myl7* promoter was chosen. Furthermore, the consistency of the phenotypes in the homozygous *tnnt2* mutant zebrafish at early developing stages also make this model ideal for quantitative analysis.

### Identification of a non-cardiomyocyte Tnnt2-positive smooth muscle cell population in zebrafish OFT

Both *tnnt2a* and *myl7* are typical cardiomyocyte-specific genes with slight differences in their expression patterns. In our study, we identified a small population of Tnnt2-positive but *myl7*-negative cells, which are indispensable to normal heart function in zebrafish. In other teleost, a smooth muscle organ called bulbous arteriosus (BA) has been previously described in the OFT, which displays contraction at each pumping (Moriyama et al., 2016). In mammals, molecular and histological studies have identified the presence of a similar structure, which is an α-SMA-positive smooth muscle system that is differentiated from heart interstitial cells (Ayoub et al., 2016; Latif et al., 2015) in the aortic valve leaflets (AVL) (Bairati and DeBiasi, 1981; Taylor et al., 2000; Rogers et al., 2003). Teleost BA and mammalian AVL in the OFT may be evolutionally conserved in function and cellular composition (Moriyama et al., 2016).

### The hints for gene therapy exclusively targeting cardiomyopathy

Current therapeutic strategies, as well as patient outcomes associated with cardiomyopathy, remain disappointing. Over 40% of children with DCM die within 2 years without a heart transplantation (Lee et al., 2017). In comparison to compound-based therapies, gene therapy holds promise for an ultimate cure for cardiomyopathy. In preclinical experiments, Jiang et al. (2013) and Mearini et al. (2014) delivered an adeno-associated virus (AAV9) carrying *Myh6* siRNA or *Mybpc3* cDNA into the thoracic cavity or temporal vein of *Myh6* or *Mybpc3* mutant mice, respectively, relieving cardiomyopathy symptoms in both models. Although the virus-based delivery techniques used in most clinical gene therapies are not commonly applied to zebrafish, the convenient transgenesis in zebrafish via zygote injection can easily simulate or mimic the fundamental rescue strategies of those gene therapies.

Theoretically, cell/tissue-specific promoters restrain the expression of transgenes at an appropriate time and space and may be utilized to optimize gene therapy. In cases of heart diseases, cardiomyocytes have been regarded as the most preferred target cells in most current gene/cell therapies. In our study, we highlighted an important warning sign for the transgenic strategy of cardiomyocyte-exclusive rescue. The *myl7* promoter-driven *tnnt2a* replenishment failed to restore heart function in *tnnt2a* mutant zebrafish, while the additional expression of *tnnt2a* in a small group of smooth muscle cells in the OFT made up for the deficiency. In successful rescue, it was more likely that *tnnt2a* supplement expression in OFT smooth muscle cells was responsible, although it was also possible that in the embryos selected for OFT expression, expression elsewhere is also induced. Our results demonstrated how slightly different expression patterns between two promoters may significantly affect the rescue efficiency of genetic replenishment, and the selection of non-endogenous cell/tissue-specific promoters for developing treatment for patients with cardiomyopathy should be carefully assessed.

## MATERIALS AND METHODS

### Zebrafish

Zebrafish were raised in a circulating water system at 28.5°C and were euthanized by bathing them in tricaine (200 mg l^−1^) for 2 min. Wild type (AB strain), Tg(*myl7*: *eGFP*) (Yao et al., 2017; Zhang et al., 2017) and Tg (*bactin2:GCamp6s*) (Liu et al., 2017), zebrafish were used as previously reported. Tg(*myl7:tetOn; tre:tnnt2a-p2A-mKate2; myl7:eGFP*); *tnnt2a*^−/−^ were generated by outcrossing Tg(*myl7:tetOn; tre:tnnt2a-p2A-mKate2; myl7:eGFP*) with *tnnt2a*^+/−^. Procedures involving animals were approved by the Fudan University Shanghai Medical School Animal Care and Use Committee and was conducted in conformity with National Institutes of Health Guidelines for the Care and Use of Laboratory Animals.

### Generation of *tnnt2a* mutant via CRISPR-Cas9 technique

To introduce mutations in *tnnt2a*, a target site was selected using the CRISPR design website (http://zifit.partners.org/ZiFiT/). Guide RNA (gRNA) expression plasmid was generated by connecting oligo to pMD18-T, which has been enzyme-digested by BbsI. Then pGH-T7-zCas9 was digested by XbaI to produce a zebrafish codon-optimized version of Cas9 mRNA. As previously described, gRNA and Cas9 mRNA were prepared via *in vitro* transcription (Jao et al., 2013). One-cell stage zebrafish embryos were microinjected with 300 ng µl^−1^ Cas9 mRNA and 30 ng µl^−1^ gRNA. Mature F_0_ zebrafish were crossbred with wild-type AB to generate offspring containing heterozygous mutants. Heterozygous mutants were incrossed to obtain homozygous mutants, and outcrossed to Tg(*myl7*: *eGFP*) or Tg(*myl7:TetOn;tre:tnnt2a-mKate2; myl7:eGFP*) for advanced studies. Genotyping was performed via PCR-sequencing.

*tnnt2a* target sequence: 5′-GGGAGCGACAGAAGCGTT-3′,

gRNA Oligo s: 5′-ataGGGAGCGACAGAAGCGTTgt-3′,

gRNA Oligo vs: 5′-taaaacAACGCTTCTGTCGCTCC-3′,

*tnnt2a* Genotyping Primer Forward: 5′-gtaagcgcatggagaaggac-3′,

*tnnt2a* Genotyping Primer Reversed: 5′-gcgacatcacagagccaaat-3′.

### Generation of Tg(*myl7:TetOn;tre:tnnt2a-p2a-mKate2*) zebrafish via Tol2 transposons

RNA was extracted from zebrafish at 3 dpf (*N*=50) and cDNA was obtained using SuperScript^®^III First-Strand Synthesis System (Invitrogen). The *tnnt2a* cDNA was successfully cloned into the PCR^®^II vector to generate pPCRII___*tnnt2a* cDNA. Knockin plasmid was constructed by attL/attR recombination (LR) reaction. Three entry clone vectors, pENTR-5′-cmlc2, pENTR-Tetone and pENTR-3′-*tnnt2a*(cDNA)-p2A-mKate2. pENTR-5′-cmlc2 were constructed from the template pDestTol2CG2 (Tol2kit). Next, pENTR-Tetone was successfully obtained via attB/attP recombination (BP) reaction, performed after overlap PCR from three templates: first template [PLVX-TetOn3G(Clontech)]; second template [p3E-polyA (Tol2kit)]; and third template [PLVX-TRE3G (Clontech)]. pENTR-3′-*tnnt2a*(cDNA)-p2A-mKate2 was also constructed via a BP reaction, operated following two-fragment overlap PCR from two templates: first template (pPCRII___*tnnt2a* cDNA) and second template (p3E-P2A-mKate2, from our lab). All primer sequences were listed in Table S4. Additionally, pDestTol2CG2 was used as LR vector. NotI digestion was used to linearize pCS2FA-transposase (Tol2kit) and transposon RNA was synthesized using mMESSAGE mMACHINE^®^ SP6 Transcription Kit (Invitrogen) and purified using PureLink^®^ RNA Mini Kit (Invitrogen). A mixture of knockin plasmid (30 ng µl^−1^) and Tol2 transposase RNA (50 ng µl^−1^) was directly injected into the one-cell-stage zygote. Successful generation of knockin zebrafish was confirmed via heart-specific green or red fluorescence with/without DOX induction. Knockin zebrafish larvae were nurtured into adulthood and outcrossed with wild-type AB or mutant zebrafish.

### Imaging and image processing

Live zebrafish larvae narcotized with 200 mg l^−1^ tricaine (Sigma-Aldrich, A5040) were mounted in 3% methylcellulose for imaging. Fixed samples were balanced and located in 80% glycerol before imaging. Images of whole zebrafish or dissected heart, and movies of the beating heart were obtained via either a fluorescent dissecting microscope (Olympus, DP73) or a confocal microscope system (Leica, TCS-SP8). All videos were taken at 10 frames per second. Confocal images are shown as single optical sections or maximum intensity Z projections, respectively, as indicated. Heart atrium and ventricle diameter measurements were performed at the end of atrial and ventricular diastole using straight measurement in ImageJ. Pericardium size was measured using area measurement in ImageJ. The maximum diameter of OFT was used to calculate the difference between diastolic and contractile diameters of OFT.

### DOX induction

Doxycycline (Sigma-Aldrich, D9891) was added to E3 medium to produce a final concentration of 80 µg ml^−1^. Zygotes were treated with DOX at 16 hpf or 48 hpf. E3 medium containing doxycycline was changed every 12 h.

### Transcriptome resequencing analysis (RNA-seq)

Zygotes were obtained by crossing Tg(*myl7:TetOn; tre:tnnt2a-mKate2; myl7:eGFP*); *tnnt2a*^+/−^ with *tnnt2a*^+/−^. DOX was added at 16 hpf to ensure full rescue of Tg(*myl7:TetOn;tre:tnnt2a-mKate2;myl7:eGFP*); *tnnt2a^−/−^*. At 48 hpf, transgene group zebrafish was screened via fluorescence. At 72 hpf, tails were cut to identify genotypes, and the body was placed in a 96-well plate filled with Trizol. Zebrafish (*N*=50 per group) with identical mutants were collected to extract RNA using the RNeasy Plus Mini Kit (Qiagen 74132). Library construction, sequencing and bioinformatics analyses were performed as previously described (Fu et al., 2013). In brief, cDNA libraries were constructed according to the manufacturer's standard protocol (Illumina, Inc.). Oligo (dT) isolated mRNA was fragmented, and cDNA was synthesized using these mRNA fragments as templates. Short fragments were purified and resolved with EB buffer for end reparation and single nucleotide adenine addition. Next, the short fragments were connected with adapters, and suitable fragments were selected for PCR amplification. During the QC steps, the Agilent 2100 Bioanaylzer and ABI StepOnePlus Real-Time PCR System were used in quantification and qualification of sample libraries. Libraries were sequenced using the Illumina HiSeq 4000 platform. After sequencing, we first filtered low-quality, adapter-polluted and high content of unknown base (N) reads to separate clean reads. The clean reads were mapped to the reference genome using HISAT. All coding transcripts were then merged with reference transcripts to get complete references. Next, we performed gene expression analysis using these references via StringTie (Kim et al., 2015) and Biowtie (Shen et al., 2014). We then detected DEGs via DESeq2 (parameters: Fold Change≥2.00 and Adjusted *P*≤0.05) and performed Gene Ontology (GO) classification and functional enrichment, as well as KEGG pathway classification and functional enrichment for DEGs using phyper, a function of R (r-project.org). Sequencing was performed once for each sample as an initial screening. The raw data may be downloaded from SRA: SRP7896793, SRP7896794, SRP7896795, and SRP7896796 (Table S3).

### Real time RT-PCR

Zebrafish larvae at 72 hpf (*N*=30 per sample, 3 samples per group) were fixed in 4% PFA at 4°C for 1 h before the hearts were dissected. Total RNA was extracted from whole zebrafish larvae or dissected hearts by using the RNeasy FFPE Kit (QIAGEN 73054). PrimeScript RT reagent kit (Takara RR037A) was used to synthesize cDNA. Real time RT-PCR was conducted using SYBR Premix Ex Taq II (Takara RR420D) in the StepONEPlus Real-Time PCR system (Applied Biosystems). The mRNA levels were normalized to an average of β-actin as an internal control. The primers used were listed in Table S5.

### *In situ* hybridization

Digoxin (Roche, 3359247910) labelled full-length *tnnt2a* mRNA probe was obtained by pPCRII___*tnnt2a* cDNA *in vitro* transcription using MAXIscript™ T7 Transcription Kit (Invitrogen, AM1314). Detailed process of whole-mount *in situ* hybridization were performed as described previously (Thisse and Thisse, 2008).

### Immunofluorescence

Zebrafish larvae were fixed in 4% PFA for 4 h at room temperature (RT). The samples were washed with PBS thrice, and digested with collagenase (Invitrogen, 17100017) for 40 min at RT. Next, samples were washed with PBS before the second fixing in 4% PFA for 20 min at RT. Antigen retrieval was performed using 2 M hydrochloric acid (10 min at RT and 20 min at 37°C), which was then neutralized via Tris-HCl (PH>8.0). The samples were blocked in PBS with 0.1% Triton X-100 and 10% normal goat serum (PBT) for 1 h at RT, incubated with primary antibody cardiac troponin T(mouse) (Genetex, 10214; 1:500), alpha smooth muscle Actin (Rabbit) (Abcam, 15734; 1:1000), myl7 antibody (Rabbit) (Genetex, 128346; 1:3000), Alexa Fluor™ 568 Phalloidin (Invitrogen), Hoechst33342 (Invitrogen, H21492; 1:1000) in PBT overnight. Goat anti-rabbit Alexa 488 (Jackson ImmunoResearch, 115-545-003; 1:500), goat anti-rabbit Alexa 594 (Jackson ImmunoResearch, 115-585-003; 1:500) and goat anti-mouse Alexa 647 (Jackson ImmunoResearch, 115-605-003; 1:500) were used as secondary antibodies. After 4 h, the sample was washed thrice in PBS before mounting and imaging.

### Rescue vector construction and designing of the double-rescue experiment

The rescue vector *elf1α: tetOn; tre:tnnt2a-p2a-mKate2* was constructed via LR reaction. Three entry clone vectors were, pENT-5′-ef1α, pENT-Tetone, and pENT-3′-*tnnt2a*(cDNA)-p2A-mKate2, where pENT-5′-ef1α was obtained from Dr Zhang (Zhang et al., 2017) and pDestTol2PA2 was used as the LR reaction vector. In double-rescue experiments, the rescue vector *elf1α: tetOn; tre:tnnt2a-2A-mKate2* zygotes were injected into the fertilized eggs obtained by crossing Tg(*myl7:tetOn; tre:tnnt2a-mKate2; myl7:eGFP*); *tnnt2a*^+/−^ with *tnnt2a*^+/−^. DOX was added at 16 hpf to ensure full double rescue of Tg(*myl7:tetOn;tre:tnnt2a-mKate2;myl7:eGFP*); *tnnt2a^−/−^*. Fluorescent transgenic markers were screened at 48 hpf, and the genotypes were identified using genomic DNA from cut tails at 72 hpf.

### Quantification and statistical analysis

Diameter measurements, statistical calculations and graphs were generated using ImageJ and GraphPad Prism 6. Data are expressed as mean±s.d. (*N* is listed in each figure legend). Statistical differences were evaluated using two-tailed unpaired *t*-test, unpaired *t*-test with Welch's correction, Kruskal–Wallis test, one-way ANOVA, and two-way ANOVA.

## Supplementary Material

Supplementary information
