# Supplementary material for: Combinatorial genetic replenishments in myocardial and outflow tract tissues restore heart function in tnnt2 mutant zebrafish
Source: Biol Open. 2019 Dec 9;8(12):bio046474. doi: 10.1242/bio.046474 (PMC6918781; doi:10.1242/bio.046474)
Supplement: Supplementary information [file biolopen-8-046474-s1.pdf]

Supplemental information

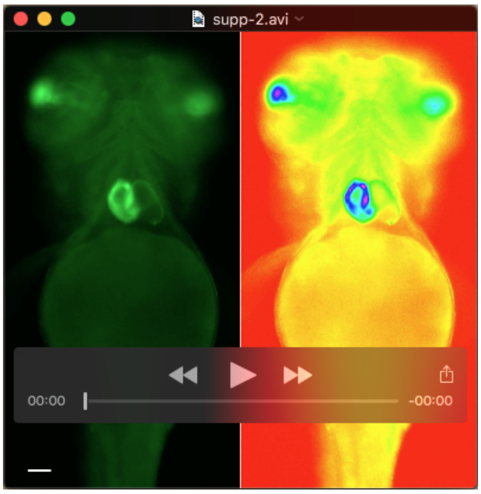

Movie 1. Time-elapse video and pseudo-colors of a 3 dpf Tg(*bactin2:GCamp6s*); *tnnt2a*$^{+/+}$ larva from the ventral view.

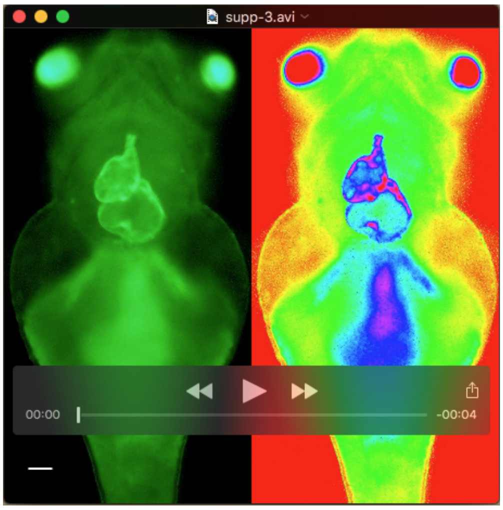

Movie 2. Time-elapse video and pseudo-colors of a 3 dpf Tg(*bactin2:GCamp6s*); *tnnt2a*$^{-/-}$ larva from the ventral view.

Biology Open • Supplementary information

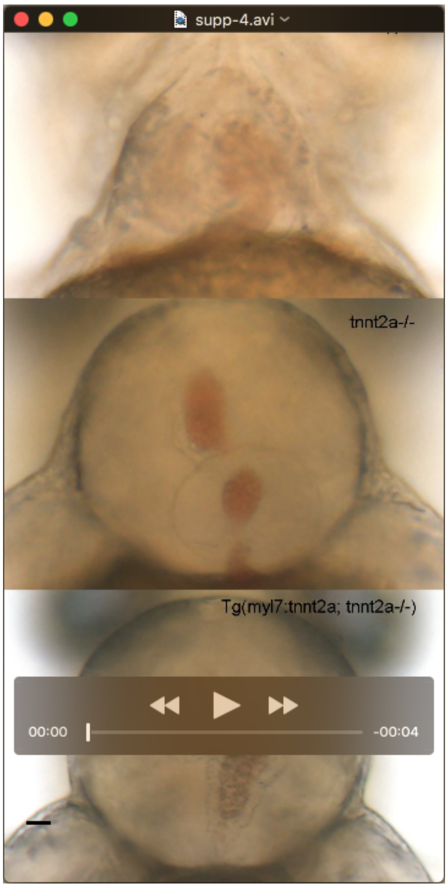

Movie 3. Bright field video of the heart regions of wildtype, *tnnt2a*$^{-/-}$, and Tg(*myl7:tnnt2a*); *tnnt2a*$^{-/-}$ zebrafish at 3 dpf.

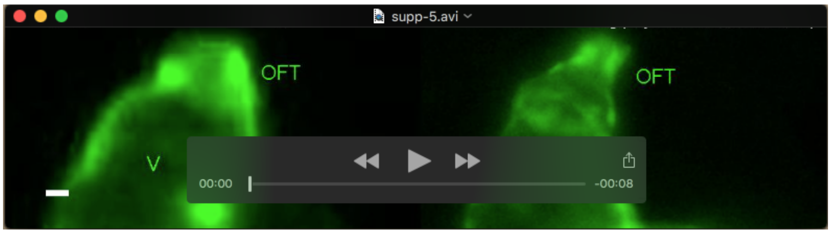

Movie 4. Time-elapse video of OFT behavior in wildtype and Tg(*myl7:tnnt2a*); *tnnt2a*$^{-/-}$ zebrafish at 3 dpf.

Biology Open • Supplementary information

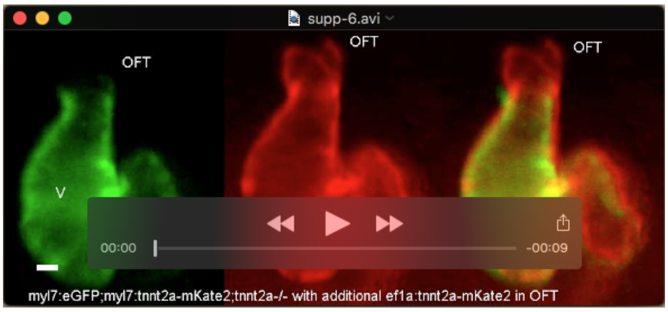

Movie 5. Time-elapse video of the heart region in a representative Tg(*myl7:tetOn; tre:tnnt2a-p2a-mKate2; myl7:eGFP*); *tnnt2a*$^{-/-}$ larvae carrying additional red fluorescence of mKate2 (and Tnnt2a) in the OFT (without overlapping green fluorescence).

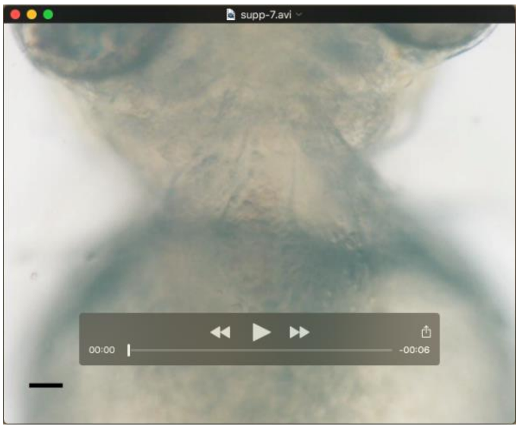

Movie 6. Bright field video of a representative double-rescued *tnnt2a*$^{-/-}$ heart that was fully functional.

Supplementary Table

Table S1. Fold changes of sarcomere and valve or OFT relative genes of wildtype and mutant zebrafish in RNA-seq

	tnnt2a$^{+/+}$	*tnnt2a*$^{-/-}$	$\log_2$FoldChange (*tnnt2a*$^{-/-}$/ *tnnt2a*$^{+/+}$)	P value	FDR
tnnt2a	7.42	0.3	-4.6283848	3.44E^{-13}	5.14E^{-12}
actc1a	354.27	397.98	0.16784663	6.54E^{-06}	4.06E^{-05}
myl7	20.89	28.05	0.42518828	0.009689	0.0284507
tnni1b	6.91	10	0.53324238	0.342696	0.4976011
tpm4a	82.32	112.57	0.45150751	1.74E^{-13}	2.66E^{-12}
tnnc1a	10.89	9.5	-0.1970045	0.221544	0.3629792
has2	1.02	0.9	-0.1805722	0.704468	0.8029169
tbx20	2.24	2.38	0.08746284	0.807426	0.8733331
gata4	2.24	7.11	1.66635083	1.1E^{-05}	6.75E^{-05}
gata5	6.83	7.19	0.07410619	0.723448	0.8153896
tbx2b	27.77	23.48	-0.2420948	0.009189	0.0271485
bmp2b	12.42	9.23	-0.4282626	0.016952	0.045948

Biology Open • Supplementary information

Table S2. Fold changes of sarcomere and valve or OFT relative genes of wildtype and transgene rescue zebrafish in RNA-seq

	tnnt2a$^{+/+}$	Tg(*myl7:tnnt2a*); *tnnt2a*$^{-/-}$	log_2FoldChange (Tg; *tnnt2a*$^{-/-}$/ *tnnt2a*$^{+/+}$)	P value	FDR
tnnt2a	7.42	5.25	-0.4991018	0.105368	0.2108288
actc1a	354.27	368.28	0.05595375	0.248902	0.40047213
myl7	20.89	21.1	0.01443051	0.980672	0.98679463
tnni1b	6.91	7.47	0.11242253	0.286924	0.44193824
tpm4a	82.32	112.5	0.45061011	$7.54E^{-13}$	$1.05E^{-11}$
tnnc1a	10.89	8.87	-0.2959979	0.468862	0.63137703
has2	1.02	0.51	-1	0.0677928	0.148043
tbx20	2.24	2.65	0.24249363	0.50083	0.65227157
gata4	2.24	7.69	1.77948487	$2.03E^{-06}$	$1.37E^{-05}$
gata5	6.83	9.1	0.41398097	0.029981	0.07629847
tbx2b	27.77	27.61	-0.0083363	0.869186	0.92671892
bmp2b	12.42	12.15	-0.0317089	0.803724	0.87933139

Table S3. Raw data of the RNA-seq in *tnnt2a*$^{+/-}$; *tnnt2a*$^{-/-}$; Tg(*myl7:tetOn;tre:tnnt2a-p2a-mKate2; myl7:eGFP*); *tnnt2a*$^{+/-}$; Tg(*myl7:tetOn;tre:tnnt2a-p2a-mKate2; myl7:eGFP*); *tnnt2a*$^{-/-}$ at 3 dpf.

Click here to Download Table S3

Biology Open • Supplementary information

Table S4. Primers using in the construction of transgenic zebrafish

Primers	Sequence
pPCRII_*tnnt2a* cDNA Forward	atgtcagacaacgaagaagt
pPCRII_*tnnt2a* cDNA Reverse	cttcctgaggcctcgcttggttct
pENTR-5'-cmlc2 Forward	aaagcttaaatcagttgtgt
pENTR-5'-cmlc2 Reverse	atggtcactgtctgctttg
pENTR-Tetone First Forward	ggggacaagtttgtacaaaaaagcaggctggaccatgtctagactggacaagagc
pENTR-Tetone First Reverse	tcttatcatgtctagatcttacccggggagcatgtcaa
pENTR-Tetone Second Forward	gacatgctccccgggtaagatctagacatgataagatacatt
pENTR-Tetone Second Reverse	aagggcctcatcgataaacttgtttattgcagctta
pENTR-Tetone Third Forward	agctgcaataaacaagtttatcgatgaggccctttcgt
pENTR-Tetone Third Reverse	ggggaccactttgtacaagaaagctgggtgcccggatccaagtataaga

Biology Open • Supplementary information

Table S5. Real time RT-PCR primers list

Gene	Forward primers	Reverse primers
tnnt2a	gcgacagaagcgtttggag*	ctgagagcagattcattggca
actc1a	tgatgagactaccgcactcg	gagcctcgtctcccacatag
myl7	gaaccgggatggagttatca	tgggtcattagcagcctctt
tnni1b	gctggcagataaggaagacg	tgaggggtaaattcaaacgc
tpm4a	tgacttctttggacgctgtg	gcggttctcgattaccttca
myh7	attttggtgccagtggaaag	cctccctctgcttctgtttg
tnnc1a	gaaccctacccctgaagagc	ttttgtctccgtccctcatc
mybpc3	ccaaggaggactgtggacat	ccagatgaatcttgggagga
has2	aggtcatgtacaccgccttc	caagggtccgctgatacatt
gata4	gctcctctgaaggtcagtcg	cagaatcaggctgttccaca
gata5	cgacaacactgtggaggaga	gtagcgccagacactgttga
gata6	tatgtcacaccgcagctctc	agacatgcgcttctgaggtt
tbx20	gtttgccaaaggcttcagag	gctgagggagaggttgtcag
tbx2a	gacgactcatcttgcgaaca	tctgcttcctttttggagga
tbx2b	gcagagatgctggtcattca	tcgtctttcttctccgcaat
tbx1	gtttgccaaaggcttcagag	ctggtgagtggggtcagaat
bmp2b	ccagcagagcaaacacgata	agccactcgtactggcatct
notch1b	cctggatgactgtcagagca	aagccattgactccatccac

***:** *tnnt2a* qPCR forward primer spans the mutation site.

Biology Open • Supplementary information

Supplementary Figures

Figure S1

A

volcano plot of 3dpf *tnnt2a*$^{-/-}$ vs. Tg(*tnnt2a*);*tnnt2a*$^{-/-}$ RNA-seq

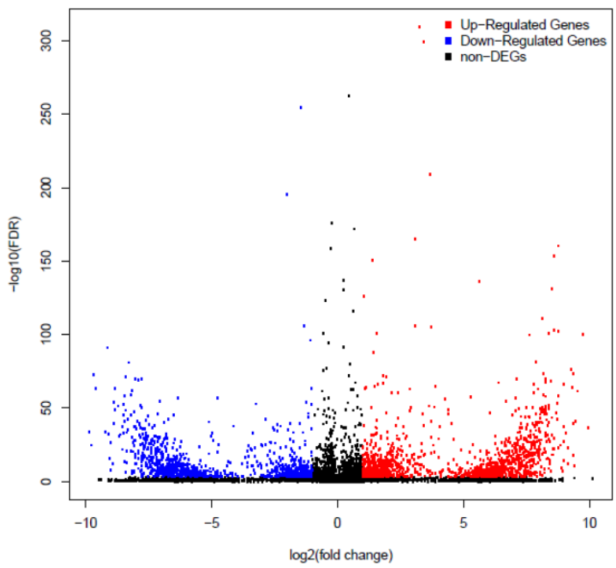

B

volcano plot of 3dpf *tnnt2a*$^{+/-}$ vs. *tnnt2a*$^{-/-}$ RNA-seq

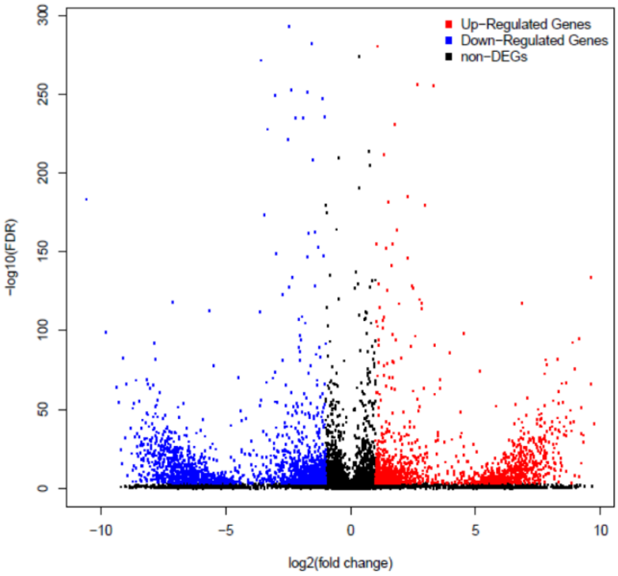

Figure S1 Volcano plot of RNA-seq

A: Volcano plot of differential expressed genes in *tnnt2a*$^{-/-}$ vs Tg(*myl7:TetOn;tre:tnnt2a-mKate2; myl7:eGFP*); *tnnt2a*$^{-/-}$

B: Volcano plot of differential expressed genes in *tnnt2a*$^{+/-}$ vs *tnnt2a*$^{-/-}$

Biology Open • Supplementary information

Figure S2

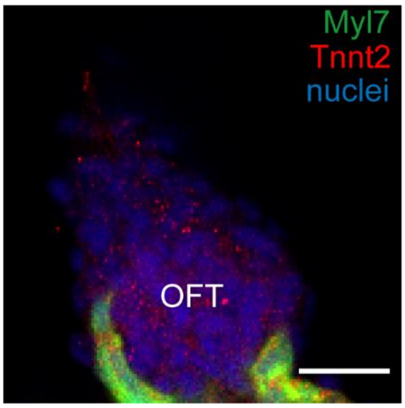

Figure S2 Cytoplasm localization of Tnnt2 in OFT

Immunofluorescence of Tnnt2 and Myl7 staining in wildtype zebrafish heart outflow tract at 5 dpf: nuclei is stained by Hoechst 33342, OFT: outflow tract; bar: 25 µm

Biology Open • Supplementary information
